# Presence of Dry Eye in Patients with Hashimoto's Thyroiditis

**DOI:** 10.1155/2014/754923

**Published:** 2014-12-14

**Authors:** Emrah Kan, Elif Kılıçkan, Gulçin Ecemiş, Emrullah Beyazyildiz, Ramis Çolak

**Affiliations:** ^1^Samsun Training and Research Hospital, Department of Ophthalmology, 55090 Samsun, Turkey; ^2^Samsun Training and Research Hospital, Department of Endocrinology and Metabolism, Samsun, Turkey; ^3^Department of Endocrinology and Metabolism, Şevk Atasagun State Hospital, Nevşehir, Turkey; ^4^Department of Endocrinology and Metabolism, Ondokuz Mayıs University, Samsun, Turkey

## Abstract

*Purpose*. To evaluate the tear function tests in patients with Hashimoto's thyroiditis and to compare the results with healthy subjects. *Methods*. A hundred and ten patients with Hashimoto's thyroiditis and 100 healthy subjects were included in this study. The presence of thyroid-associated ophthalmopathy and tear function tests were evaluated clinically. The results were first compared between the patients and the control groups and then compared between patients with NOSPECS and patients without NOSPECS. Logistic regression analyses of the risk factors for dry eye including sex, gender, free plasma thyroxine, proptosis, upper eyelid margin-reflex distance, and duration of the disease were also evaluated. *Results*. The mean ocular surface disease index score was significantly higher and mean Schirmer and mean tear break-up time scores were significantly lower in patients compared to control subjects. Mean Schirmer and tear break-up time scores were found to be significantly lower in patients with NOSPECS when compared to the patients without NOSPECS. Both proptosis and free plasma thyroxine levels were significantly associated with dry eye. *Conclusions*. Patients with Hashimoto's thyroiditis tend to develop dry eye more common than healthy subjects. Proptosis and lower free plasma thyroxine levels were found to be risk factors for the presence of dry eye.

## 1. Introduction

Thyroid-associated ophthalmopathy (TAO) is an autoimmune disorder of the extraocular muscles and surrounding orbital connective tissue including lacrimal gland which is generally associated with Graves' disease (GD) and rarely accompanies Hashimoto's thyroiditis [1]. The characteristic clinical findings of the TAO include proptosis due to an increase in the retroorbital soft tissue, lid retraction, restrictive extraocular myopathy, optic neuropathy, and inflammatory ocular surface disorders [2]. Dry eye is the most frequent cause of the ocular discomfort in TAO and has been found to be present in 85% of patients, yet the etiopathogenesis remains unclear [3]. T-cell-dependent inflammation of the ocular surface and increased tear film evaporation and osmolarity due to lid retraction and exophthalmus play an important role in ocular surface drying [4–6]. Besides, it has been shown that lacrimal gland is a target organ for thyroid hormone which expresses thyroid hormone receptor *β*-1 (Thrb). Chronically reduced thyroid hormone levels were found to modulate the expression of Thrb in lacrimal gland, thus causing a decrease in tear production and subsequent dry eye in experimental studies [7]. Although most of the studies showed the presence of dry eye in Graves' disease, it has been rarely studied in Hashimoto's disease. That is why in the recent study we aimed to evaluate the presence of dry eye in Hashimoto's disease and to compare with control subjects.

## 2. Subjects and Methods

A hundred and ten randomly selected patients with Hashimoto's thyroiditis who were referred from the endocrinology clinic were included in the study. The control group consisted of 100 healthy subjects. The local ethics committee's approval was received for the study and informed consent of the participating subjects was obtained. Patients were excluded from the study if they had any other ophthalmic disorder, had undergone any ophthalmic surgery, or had any additional systemic disease or radioactive iodine treatment within the prior 1 year.

The control subjects were chosen randomly among the patients who attended the clinic for refraction error and had no symptoms of any ocular inflammation. Neither the patients nor the control subjects had used any topical medications. The diagnosis of Hashimoto's thyroiditis was based on standard clinical criteria and confirmed by thyroid function testing and thyroid antibody tests [8]. The laboratory findings of patients and controls, including free plasma thyroxine (fT4) and thyroid stimulating hormone (TSH) levels, are summarised in Table 1.

All patients and control subjects were evaluated by a single experienced ophthalmologist for the presence of ophthalmopathy and dry eye. The diagnosis of ophthalmopathy was based mainly on the clinical state (eyelid retraction, periorbital swelling, diplopia, and others). The grade, severity, and activity of the cases were classified according to the NOSPECS classification [9]. The lid retraction was assessed by measuring the upper eyelid margin-reflex distance (UER), which is the distance between the centre of the pupillary light reflex and upper eye lid margin in primary gaze position. A measurement of 3–5 mm is considered as normal and measurement greater than 5 mm was considered as UER. According to Hertel measurements, difference of >2 mm between two eyes or proptosis of >20 mm was accepted as significant proptosis. The ocular surface disease index (OSDI), Schirmer tear test (without topical anesthesia), and tear break-up time (TBUT) were performed in all patients and the control subjects.

### 2.1. The OSDI Questionnaire

The OSDI was developed to measure the severity of dry eye, according to the OSDI questionnaire. It consists of 12 questions each scored by the patient. It has been used to evaluate the severity of the symptoms and response to the treatment in dry eye patients. OSDI subscale scores can range from 0 to 100, where 0 indicates no disability and 100 indicates complete disability [10].

### 2.2. Schirmer Tear Test

Tear secretion was measured by the Schirmer tear test (without topical anesthesia) in each eye of the patients and the control subjects. A standard Schirmer test strip was placed in the lower fornix at the junction of the lateral and middle third. After 5 min, the strips were removed and the wetted length of the test strip was measured in millimeters to determine the Schirmer test value. Aqueous tear deficiency was defined as a Schirmer test value without topical anesthesia of less than 5 mm at 5 minutes.

### 2.3. Tear Break-Up Time

To measure TBUT a fluorescein sodium strip moistened with a drop of nonpreserved saline solution was applied to the inferior palpebral conjunctiva in each eye of the patients and the control subjects. After removing the strip, the patient was asked to blink three times and then look straight forward. The precorneal tear film was examined with a biomicroscope and the elapsed time before the initial break-up, rupture of the tear film was recorded. The TBUT was measured three times and the measurements were averaged.

### 2.4. Statistical Analysis

Statistical analysis was performed using the Statistical Package for the Social Sciences version 13.0 (SPSS, Chicago, IL, USA). Pearson's Chi-square test was used for the comparison of categorical variables between patients and controls and Student's *t*-test was used for the comparison of continuous variables between groups. Finally, a *P* value < 0.05 was considered as statistically significant. Logistic regression analysis was also used to determine the significant risk factors for dry eye.

## 3. Results

Characteristics of Hashimoto's thyroiditis patients and control groups are shown in Table 2. Sex and age prevalence were similar between the two groups. The mean time of the disease was 39.3 months (between 4 and 180 months). Dry eye symptoms were significantly higher in the patient group when compared to the controls. The mean OSDI score in the patient group was 32.7 ± 19.2 and it was 18.9 ± 14.8 in the control group (*P* = 0.001). The mean Schirmer tear test score was 10.9 ± 5.6 mm and 17.7 ± 6.8 mm in the patient and control groups, respectively. The difference was statistically significant (*P* = 0.001). The mean TBUT in the patient group was 10.1 ± 2.6 sec which was significantly increased in the control group to 13.2 ± 3.2. In control patients one person had proptosis and another had lid retraction.

We also made logistic regression analyses of the risk factors for dry eye including sex, gender, fT4, proptosis, UER, and duration of the disease in patients with Hashimoto's thyroiditis. The logistic regression analysis showed that both proptosis and fT4 levels were significantly associated with dry eye (Table 3). There was a negative correlation between fT4 levels and dry eye; that is, lower levels were associated with more severe dry eye. According to the NOSPECS classification, 13 patients (11.8%) had UER, 12 had soft tissue involvement (oedema of conjunctivae and lids, conjunctival injection, etc.) (10.9%), 7 had proptosis (6.4%), and 6 had extraocular muscle involvement (5.5%), whereas no patients had corneal or optic nerve involvement. Of these clinically active patients, apart from higher OSDI score, mean Schirmer tear test score and mean TBUT were found to be significantly decreased when compared to the patients not entering this classification (Table 4).

## 4. Discussion

It has been demonstrated in this study that dry eye was found to be more common in patients with Hashimoto's thyroiditis than control subjects and both proptosis and deficiency of fT4 were found as risk factors for the presence of dry eye in logistic regression analyses. Over the past years, several studies have demonstrated the association of dry eye with thyroid disorders. Dry eye is a common finding and was found to be present in 85% of TAO patients [3].

Numerous studies have investigated the pathogenesis of dry eye associated with TAO. One of the suggested mechanisms is that increased lid fissure width and proptosis due to increased orbital volume in TAO accelerate tear film evaporation and increase tear film osmolarity. Increased tear film osmolarity has been reported to stimulate the production of inflammatory factors, such as interleukin- (IL-) 1s, tumor necrosis factor- (TNF-) *α*, and matrix metalloproteinase- (MMP-) 9. The mitogen-activated protein kinase (MAPK) signaling pathways in the ocular surface epithelial cells were also activated by tear film hyperosmolarity. The activation of MAPK signaling pathways is known to stimulate the expression of MMP-9 and the production of inflammatory cytokines, which then lead to ocular surface damage and dry eye [11, 12]. Gilbard and Farris reported that the tear osmolarity of patients with thyroid eye disease was abnormally high [5]. Our results showed that degree of proptosis was significantly higher in the patients with Hashimoto's thyroiditis than in the healthy subjects. The TBUT was significantly lower in patients with Hashimoto's thyroiditis suggesting an unstable tear film. We believe that increased width of the palpebral fissure which results from the proptosis in patients may have an impact on ocular surface drying and tear hyperosmolarity. Hyperosmolarity is not the sole mechanism and inflammation may have a possible effect for the development of dry eye in TAO patients as well. In a study, dry eye symptoms and findings in GD patients were compared to those in healthy controls. Although the mean palpebral fissure height and the amount of proptosis did not statistically differ between the patients and the control subjects, a high incidence of grade 2-3 metaplastic changes and high numbers of lymphocytes were found in temporal interpalpebral conjunctiva of patients when compared to the controls indicating ocular surface inflammation. As a result it was suggested that ocular surface inflammation, apart from evaporative dry eye, plays an important role in the pathogenesis of dry eye in GD [13]. In our study, we evaluated 21 patients, classified in NOSPECS classification, who had worse scores in both tear film functions and OSDI scores than other patients suggesting the role of ocular surface inflammation in pathogenesis of dry eye.

Studies that investigated the presence of dry eye in TAO have been carried out in GD patients so far. In this study we investigated whether fT4 deficiency could have an effect in the presence of dry eye in patients with Hashimoto's thyroiditis. As recent studies have demonstrated that the deficiency of thyroid hormone might predispose to ocular surface structural changes and dry eye, a study showed that lacrimal gland is a target organ for thyroid hormone and expresses thyroid hormone receptor *β*-1 (Thrb). Chronically reduced thyroid hormone levels were found to modulate the expression of Thrb in lacrimal gland and can cause dry eye [4]. In our study we included both new and old diagnosed patients to the study and hormone replacement therapy has been administered to clinic patients with Hashimoto's thyroiditis as soon as they were referred to endocrinology clinic. On top of that we do not know the inception of the disease and the deficiency of thyroid hormone before the diagnosis. However, we performed replacement therapy in clinic patients with Hashimoto's thyroiditis, considering that probable chronic insufficient fT4 levels before treatment might have an effect for the presence of decreased tear functions.

To our knowledge our study is the first study which evaluated tear function test in patients with Hashimoto's thyroiditis. We found that patients with Hashimoto's thyroiditis tend to develop dry eye more commonly than healthy subjects and dry eye is more prevalent in patients with active TAO. Proptosis is found to be a risk factor supporting the previous studies that increased palpebral fissure width might lead to ocular surface drying and tear film hyperosmolarity. The weakness of our study is that we did not measure tear osmolarity. However we suggest that decreased TBUT might be owing to hyperosmolarity caused by proptosis. In addition we showed that serum fT4 levels might have a protective effect for dry eye; hence hormone replacement therapy may have a positive effect in order to ameliorate dry eye symptoms in patients with Hashimoto's thyroiditis. As far as we are concerned, endocrinologists should be warned about this situation and tear function tests should be performed in order not to omit the dry eye and if dry eye is examined, artificial tear drops and modifications of environmental conditions should be recommended to these patients. Further studies are needed which prove hyperosmolarity by measuring the tear osmolarity and ocular surface inflammation by conjunctival biopsies.

## Figures and Tables

**Table 1:** The laboratory findings of patients and control subjects.

	fT4 (ng/dL)	TSH (mIU/mL)
	Median (min–max)	Median (min–max)
Patients (*n* = 110)	1.17 (0.06–2.30)	3.5 (0.02–99)
Healthy subjects (*n* = 100)	1.2 (0.90–1.60)	1.3 (0.3–4.0)

fT4: free plasma thyroxine; TSH: thyroid stimulating hormone.

**Table 2:** Characteristics of patients and healthy subjects.

	Patients	Healthy subjects	*P*
	(*n* = 110)	(*n* = 100)	
Age (mean ± SD)	42 ± 11	41 ± 11	0.92
Sex (M/F)	7/103	5/95	0.6
UER (%)	11.8	1	0.03^*^
Proptosis (%)	6.4	1	0.22
OSDI (mean ± SD)	32.7 ± 19.2	18.9 ± 14.8	<0.05^*^
Schirmer (mean ± SD)	10.9 ± 5.6	17.7 ± 6.8	<0.05^*^
TBUT (mean ± SD)	10.1 ± 2.6	13.2 ± 3.2	<0.05^*^

^*^Statistically significant.

UER: upper eyelid retraction, TBUT: tear break-up time, OSDI: ocular surface disease index, M: male, and F: female.

**Table 3:** Outcomes of binary logistic regression analysis.

	OR	95% CL
fT4 (ng/dL)	0.03	0.003–0.289
Proptosis	1.25	1.02–1.54

OR: odds ratio; fT4: free plasma thyroxine.

**Table 4:** Comparison of dry eye between patients with and without NOSPECS classification.

	Patients with NOSPECS (*n* = 89) (mean ± SD)	Patients without NOSPECS (*n* = 21) (mean ± SD)	*P*
OSDI	33.3 ± 19.2	32.6 ± 19.4	0.8
Schirmer	7.4 ± 4.3	11.7 ± 5.6	0.01^*^
TBUT	8.3 ± 3.3	10.5 ± 3.2	0.009^*^

^*^Statistically significant. OSDI: ocular surface disease index; TBUT: tear break-up time.
